# Zebrafish Chemical Screening Reveals the Impairment of Dopaminergic Neuronal Survival by Cardiac Glycosides

**DOI:** 10.1371/journal.pone.0035645

**Published:** 2012-04-26

**Authors:** Yaping Sun, Zhiqiang Dong, Hadie Khodabakhsh, Sandip Chatterjee, Su Guo

**Affiliations:** 1 Department of Bioengineering and Therapeutic Sciences, Programs of Human Genetics and Biological Sciences, University of California San Francisco, San Francisco, California, United States of America; 2 The George Washington University School of Medicine, Washington, D.C., United States of America; 3 The Scripps Research Institute, La Jolla, California, United States of America; Imperial College London, United Kingdom

## Abstract

Parkinson's disease is a neurodegenerative disorder characterized by the prominent degeneration of dopaminergic (DA) neurons among other cell types. Here we report a first chemical screen of over 5,000 compounds in zebrafish, aimed at identifying small molecule modulators of DA neuron development or survival. We find that Neriifolin, a member of the cardiac glycoside family of compounds, impairs survival but not differentiation of both zebrafish and mammalian DA neurons. Cardiac glycosides are inhibitors of Na^+^/K^+^ ATPase activity and widely used for treating heart disorders. Our data suggest that Neriifolin impairs DA neuronal survival by targeting the neuronal enriched Na^+^/K^+^ ATPase α3 subunit (ATP1A3). Modulation of ionic homeostasis, knockdown of p53, or treatment with antioxidants protects DA neurons from Neriifolin-induced death. These results reveal a previously unknown effect of cardiac glycosides on DA neuronal survival and suggest that it is mediated through ATP1A3 inhibition, oxidative stress, and p53. They also elucidate potential approaches for counteracting the neurotoxicity of this valuable class of medications.

## Introduction

Since its discovery as a prominent chemical neurotransmitter in the vertebrate nervous system, dopamine (DA) is recognized to have many important physiological functions including the control of movement, cognition, affect, as well as neuroendocrine secretion [1], [2]. Among various human disorders involving the dysregulation of DA systems, Parkinson's disease (PD) has the best-characterized pathology of DA neurons: the degeneration of substantia nigral DA neurons is the major cause of this most common movement disorder (second most common neurodegenerative disorder after the Alzheimer's disease). Recent studies show that neurodegeneration in PD appears more widespread than only affecting substantia nigral DA neurons, which possibly underlies some of the non-motor symptoms of the disease [3]. Since its introduction almost 50 years ago, L-DOPA, among other DA replacement therapies, remain the mainstream symptomatic treatments for PD, despite observations that they exert poor control over non-motor symptoms, and moreover, that their prolonged use leads to debilitating side effects [4]. Thus, a better understanding of both the genetic and environmental causes of PD will facilitate the development of new treatments with neuroprotective or disease-modifying effects.

DA neurons exhibit overall conserved organization and function across vertebrates [5]. In developing zebrafish embryos, DA neurons are detected in the ventral forebrain (posterior tuberculum and hypothalamus), dorsal forebrain (telencephalon), olfactory bulb and retina [6], [7] in patterns that closely resemble those found in the adult zebrafish brain [8]. These neurons display adult-like ascending and descending projections shortly after hatching [9], [10]. While DA neurons are conspicuously absent from the ventral midbrain, the ventral forebrain DA neurons ascending to the striatum (where ventral midbrain DA neurons in mammals project) are likely the functional counterpart of the mammalian midbrain DA neurons [11], [12].

Zebrafish is a vertebrate model organism that is highly amenable to chemical genetic studies [13], [14]. Owing to its high fecundity and the small size of embryos and larvae that can be fit into 96-well plates, zebrafish is particularly suitable for high content small molecule screening *in vivo*. Such screens can utilize the transparency of zebrafish embryos and larvae to monitor perturbation of distinct molecular markers and cell types in the context of an entire organism. Chemicals identified from small molecule screens are important pharmacological tools for probing the underlying biological mechanisms of disease models and can serve as leads for developing new therapeutics. Assays employing wild-type, mutants, or transgenic zebrafish embryos and larvae have led to discoveries of small molecules that regulate major signaling pathways [15], [16], cellular proliferation/differentiation [17]–[19], developmental toxicity [20], [21], disease remodeling [22], [23], or simple forms of behavior [24], [25].

Despite the functional significance of DA neurons, no small molecule screen for modulators of DA neuron development or survival has been reported. In this study, we report a first *in vivo* small molecule screen in zebrafish with the goal of identifying chemical regulators of DA neuron development or survival. We uncover a previously unknown neurotoxic effect of cardiac glycosides on DA neurons, and identify approaches to counteract the neurotoxicity of this class of medications.

## Results

### Chemical screening identifies Neriifolin, which disrupts the pattern of DA neurons in the ventral forebrain

**Figure 1:**
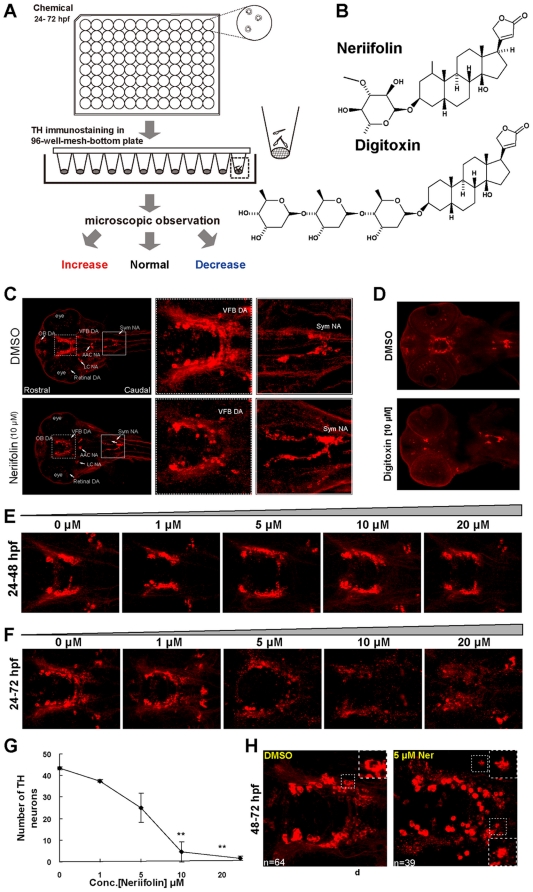
Zebrafish chemical screen identifies Neriifolin, a member of cardiac glycoside family, which disrupts the pattern of DA neurons in the ventral forebrain. (A) Schematic diagram of the chemical screening platform, through which Neriifolin was identified as a hit that decreases ventral forebrain DA neurons. (B) Structure of two cardiac glycosides, Neriifolin and Digitoxin, both of which disrupt the pattern of VFB DA neurons. (C) Embryos treated with 10 µM Neriifolin showed a decrease of VFB DA neurons (middle panels), whereas the Sym NA neurons were normal (right panels). (D) Treatment with another cardiac glycoside, Digitoxin, similarly decreased VFB DA neurons but not Sym NA neurons. (E) Embryos treated with different concentrations of Neriifolin from 24 hpf to 48 hpf showed no obvious defect in the pattern of VFB DA neurons. (F) Embryos treated with different concentrations of Neriifolin from 24 hpf to 72 hpf displayed impaired DA neuron pattern in VFB. The dose response curve is shown in (G). (H) Embryos treated with Neriifolin from 48–72 hpf also showed deficit in VFB DA neurons: neuronal numbers in the control vs. treated embryo are 64 and 39 respectively. The insets show enlarged views of DA neurons, which reveal the presence of TH in the nucleus, indicating a loss of nuclear membrane integrity. OB, olfactory bulb; VFB, ventral forebrain; sym NA, sympathetic NA neurons; AAC NA, arch-associated NA; LC, locus coeruleus.

In order to identify small molecule regulators of DA neuron development or survival, we established a chemical screening platform employing embryonic and larval zebrafish. We treated dechorionated wild-type embryos in 96-well plates (3 embryos per well) with chemicals for 48 hours (from 24–72 hours post fertilization, hpf), at a final concentration of 10 µM. At 72 hpf, embryos were fixed and stained with antibodies against tyrosine hydroxylase (TH), the rate-limiting enzyme in dopamine synthesis and an established marker for DA and NA neurons. The pattern of DA neurons were then visually analyzed and compared to vehicle control (1% DMSO) (**Fig. 1A**). The ventral forebrain (VFB) DA neurons were the focus of our analysis because of their prominence and similarity to mammalian midbrain DA neurons that degenerate in PD.

We screened two libraries: the Microsource library (containing 1520 FDA-approved compounds) and the Celera_IFLabs library (containing 3500 compounds that are predicted to target kinases), totaling 5020 compounds. 19 chemicals (0.3%) were identified that altered the pattern of VFB DA neurons following treatment. After further analyses, 17 compounds were deemed to have a general effect on embryonic development that in turn affected VFB DA neurons and were not pursued, thereby leaving 2 compounds (0.03%) that affected VFB DA neuronal patterns without grossly affecting embryonic development. One compound from the Celera library increased VFB DA neurons. However, due to its unknown mechanism of action, it will not be further discussed here. The other compound named Neriifolin (**Fig. 1B**) was particularly interesting to us: while it decreased the VFB DA neurons, TH-positive NA neurons in the sympathetic ganglia were unaffected (**Fig. 1C**).

Neriifolin is a member of the cardiac glycoside family of compounds that are widely used for treating heart disorders. To determine whether the observed effect of Neriifolin on VFB DA neurons is a characteristic of cardiac glycosides, we tested the effect of another cardiac glycoside, Digitoxin (**Fig. 1B**). When embryos were treated with Digitoxin at 10 µM, the number of VFB DA neurons was also decreased (**Fig. 1D**), suggesting that cardiac glycosides as a class of drugs have similar effects disrupting the patterns of VFB DA neurons.

### Neriifolin impairs the survival, not the differentiation of VFB DA neurons

To discern whether Neriifolin impairs the specification/differentiation or the survival of DA neurons, we treated embryos at earlier stages, from 24 hpf to 48 hpf, with 4 different concentrations of the drug: 1, 5, 10 and 20 µM. None of these treatments caused any obvious defect in the pattern of VFB DA neurons (**Fig. 1E**). In contrast, when embryos were treated for longer time (from 24–72 hpf), they displayed a dose-dependent deficit in the VFB DA neurons (**Fig. 1F and 1G**). This was not simply due to prolonged drug treatment, since embryos treated from 48–72 hpf also suffered a similar deficit of DA neurons (**Fig. 1H**): TH staining was cytoplasmic in control but appeared to fill the entire cell in Neriifolin-treated embryos, indicating the lost integrity of the nuclear membrane. These data suggest that Neriifolin impairs the survival rather than the specification or differentiation of VFB DA neurons.

In addition to affecting VFB DA neurons, we noted a dose-dependent effect of Neriifolin on embryonic morphology. At high concentrations (10 µM or higher), Neriifolin induced heart edema, dorsal curvature of the body axis, and opaqueness in the brain that is suggestive of overt neuro-degeneration. These observations suggest that other cell and organ types than VFB DA neurons can also be affected by the drug.

### Na^+^/K^+^ ATPase α3 subunit (ATP1A3) is a likely target of Neriifolin in DA neuronal death

Cardiac glycosides inhibit the activity of Na^+^/K^+^ ATPases. In addition, they also affect growth factor receptor signaling, MAP kinase, IP3, calcium, and NF-kB signaling [26]. The beneficial effect of cardiac glycosides on heart function is through binding to and inhibiting Na^+^/K^+^ ATPases, which drive ATP-dependent active transport of potassium (K^+^) ions into cells and sodium (Na^+^) ions out of cells. Na^+^/K^+^ ATPases are oligomers composed of at least two polypeptides: the α-subunit and the β-subunit. The α subunit is the catalytic subunit and contains an evolutionarily conserved cardiac glycoside-binding pocket. In mammals, the α-subunit exists as 4 isoforms (α1–α4) with distinct tissue-specific expression. Among these 4 isoforms, three (*atp1a1, 2, 3*) are expressed in the nervous system with *α3* being the most prominent one [27]. In zebrafish, ten genes encoding the α subunit have been identified, and they exhibit distinctive expression patterns [28], [29].

**Figure 2:**
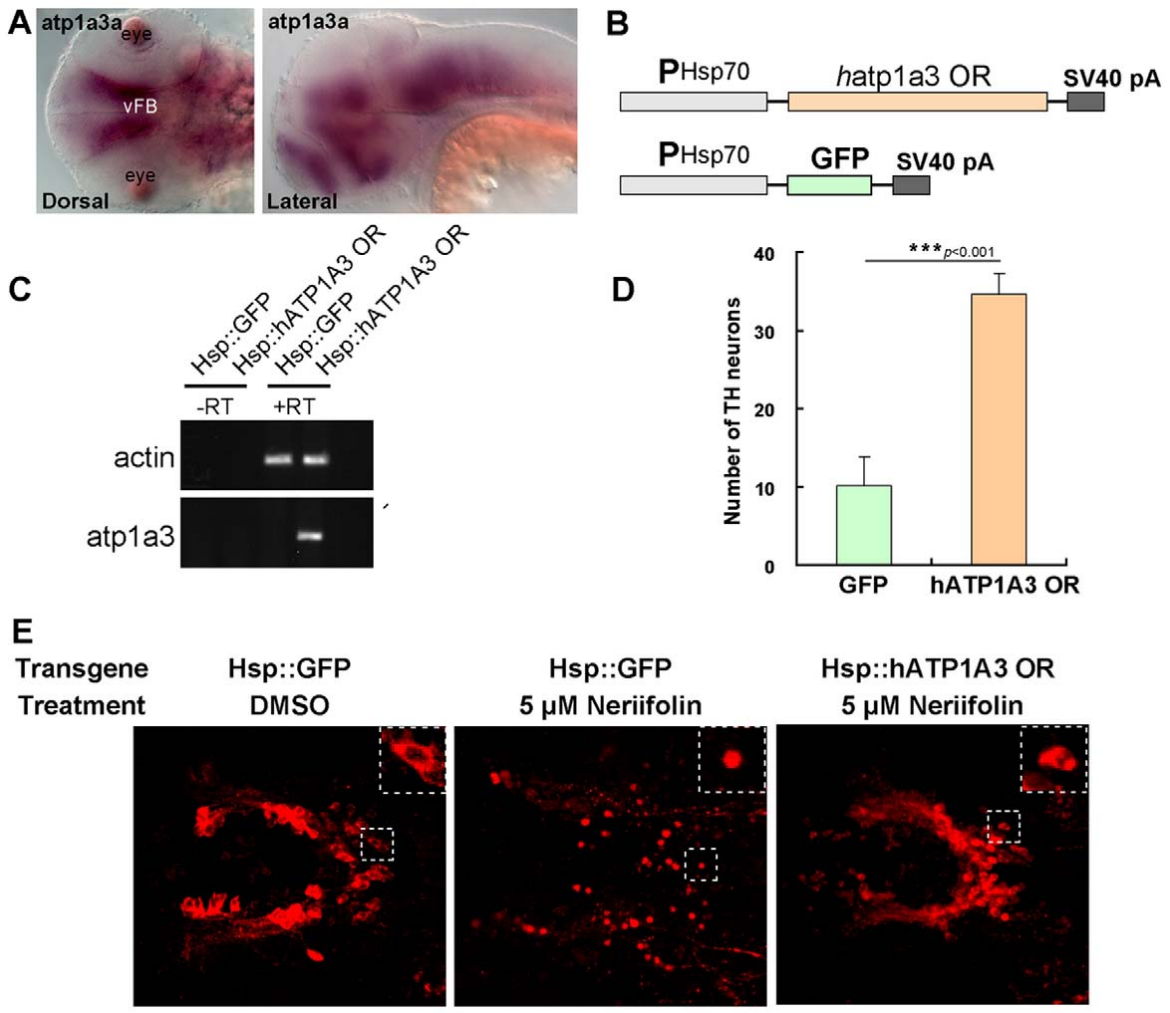
Human *atp1a3* rescues DA neurons in Neriifolin-treated embryos. (A) The expression pattern of *atp1a3a* in wild-type embryos at 48 hpf. (B) The schematic diagram of the plasmid constructs used for the rescue experiments in zebrafish embryos. (C) RT-PCR detection of the expression of human *atp1a3* in zebrafish embryos after injection and heat shock. (D–E) Quantification (D) and representative images (E) of VFB DA neurons in 5 µM Neriifolin-treated embryos that express either GFP or human *atp1a3*. Data are the averages ± SEM from 9 embryos in a single experiment that was repeated twice with similar results.

We found that the *atp1a3a* gene was prominently expressed in the ventral brain region that encompassed the VFB DA neurons (**Fig. 2A**). To determine the potential involvement of *atp1a3* in DA neuronal survival, we tested whether overexpression of the gene could rescue Neriifolin-induced DA neuronal death. Since zebrafish *atp1a3a* gene is highly homologous to the human *atp1a3* [30], we expressed the human *atp1a3* gene under the control of the heat shock-inducible *hsp70* promoter (**Fig. 2B**). This version of the human *atp1a3* gene carries two mutations (Q108R and N119D) in the cardiac glycoside-binding pocket that are known to confer resistance to Ouabain, another member of cardiac glycoside family, hence cannot be inhibited by cardiac glycosides including Neriifolin but otherwise is a fully functional Na^+^/K^+^ ATPase [30]. We injected this Ouabain-resistant form of *atp1a3* (*hsp70-ATP1A3 OR*) or the control construct (*hsp-GFP*) into one-cell stage wild-type zebrafish embryos, and performed a 90-minute heat shock from 22.5 hpf to 24 hpf. The heat-shocked embryos were subsequently treated with Neriifolin for 24 hours (from 48 hpf to 72 hpf). RT-PCR analysis confirmed the expression of human *atp1a3* in the experimental but not the control embryos (**Fig. 2C**). In the embryos injected with *hsp70-ATP1A3 OR*, Neriifolin-induced DA neuronal death was largely abrogated: most of the VFB DA neurons were healthy, whereas DA neurons in control embryos (injected with Hsp70-GFP and heat shocked) remained defective with signs of cell shrinkage, membrane fragmentation or disintegration (**Fig. 2D–E**). This data suggest that Neriifolin impairs VFB DA neuronal survival by interfering with Na^+^/K^+^ ATPase activity.

### Modulating K^+^/Na^+^ ionic homeostasis is neuroprotective against Neriifolin-induced DA neuronal death

**Figure 3:**
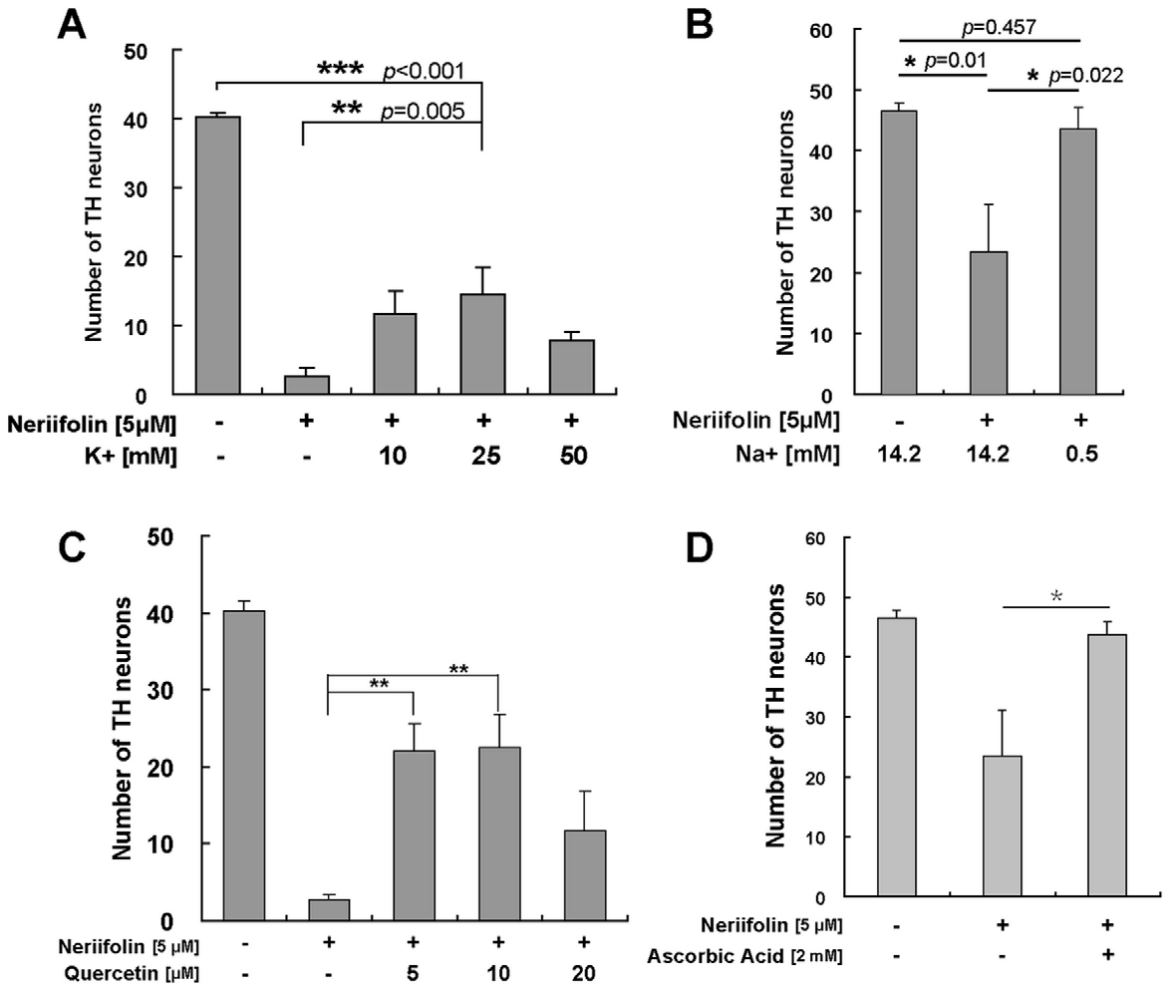
Mechanisms and protective strategies for Neriifolin-induced DA neuronal death. (A) Treatment with Neriifolin in the presence of high K^+^ in the medium significantly decreased DA neuronal loss compared to the treatment with Neriifolin alone. Data are the averages ± SEM from 6 embryos in a single experiment that was repeated twice with similar results. (B) Treatment with Neriifolin in the presence of low Na^+^ in the medium significantly decreased DA neuronal loss compared to the treatment with Neriifolin alone. Data are the averages ± SEM from 8 embryos in a single experiment that was repeated twice with similar results. (C–D) Treatment with either Quercetin (C) or Ascorbic Acid (D) significantly decreased DA neuronal death compared to treatment with Neriifolin alone. Data are the averages ± SEM from 8 embryos in a single experiment that was repeated twice with similar results.

To understand the contribution of K^+^ or Na^+^ to Neriifolin-induced DA neuronal death, we modulated their concentrations in the embryonic medium by increasing [K^+^] and/or decreasing [Na^+^]. Treatment with Neriifolin in the presence of high K^+^ (25 mM as compared to 0 mM in control) in the medium significantly protected against DA neuronal loss compared to the treatment with Neriifolin alone (**Fig. 3A**, *p* = 0.005). However, Neriifolin-induced DA neuronal death was only partially rescued, as a significant difference remained between the DMSO group and the Neriifolin/high K^+^ group (**Fig. 3A**, *p*<0.001).

Interestingly, when embryos were treated with Neriifolin in the low Na^+^ medium (0.5 mM as compared to 14.2 mM in control), the phenotype caused by Neriifolin was completely rescued: significant differences were observed between (DMSO) – (Neriifolin) groups (*p* = 0.01) and (Neriifolin) – (Neriifolin low Na^+^) groups (*p* = 0.022), but there was no difference between (Neriifolin low-Na^+^) – (DMSO) groups (*p* = 0.475) (**Fig. 3B**). These results suggest that Neriifolin-induced DA neuronal death is to a larger extent caused by the accumulation of Na^+^ rather than the depletion of K^+^. Thus, low sodium/high potassium exerts a neuroprotective effect.

### Two small molecule antioxidants, Quercetin and Ascorbic Acid, are neuroprotective against Neriifolin-induced DA neuronal death

To further understand Neriifolin-induced DA neuronal death and identify small molecule compounds that may be neuroprotective, we tested two natural food-derived compounds: Quercetin (a flavonoid abundantly present in fruits and vegetables) and Ascorbic Acid (Vitamin C). Both compounds have antioxidant properties and exert a neuroprotective effect against oxidative stress-induced neurodegeneration [31], [32]. Neriifolin-induced DA neuronal death was significantly alleviated by the treatment with either compound: significant differences were observed between the Neriifolin control and Neriifolin/Quercetin (**Fig. 3C**, *p*<0.01) or Neriifolin/Ascorbic Acid groups (**Fig. 3D**, *p*<0.05). These results suggest that Neriifolin-induced DA neuronal death is in large part due to aggravated oxidative stress, which may be caused by ionic imbalance due to ATP1A3 inhibition.

### Inhibition of p53 is neuroprotective against Neriifolin-induced DA neuronal death

**Figure 4:**
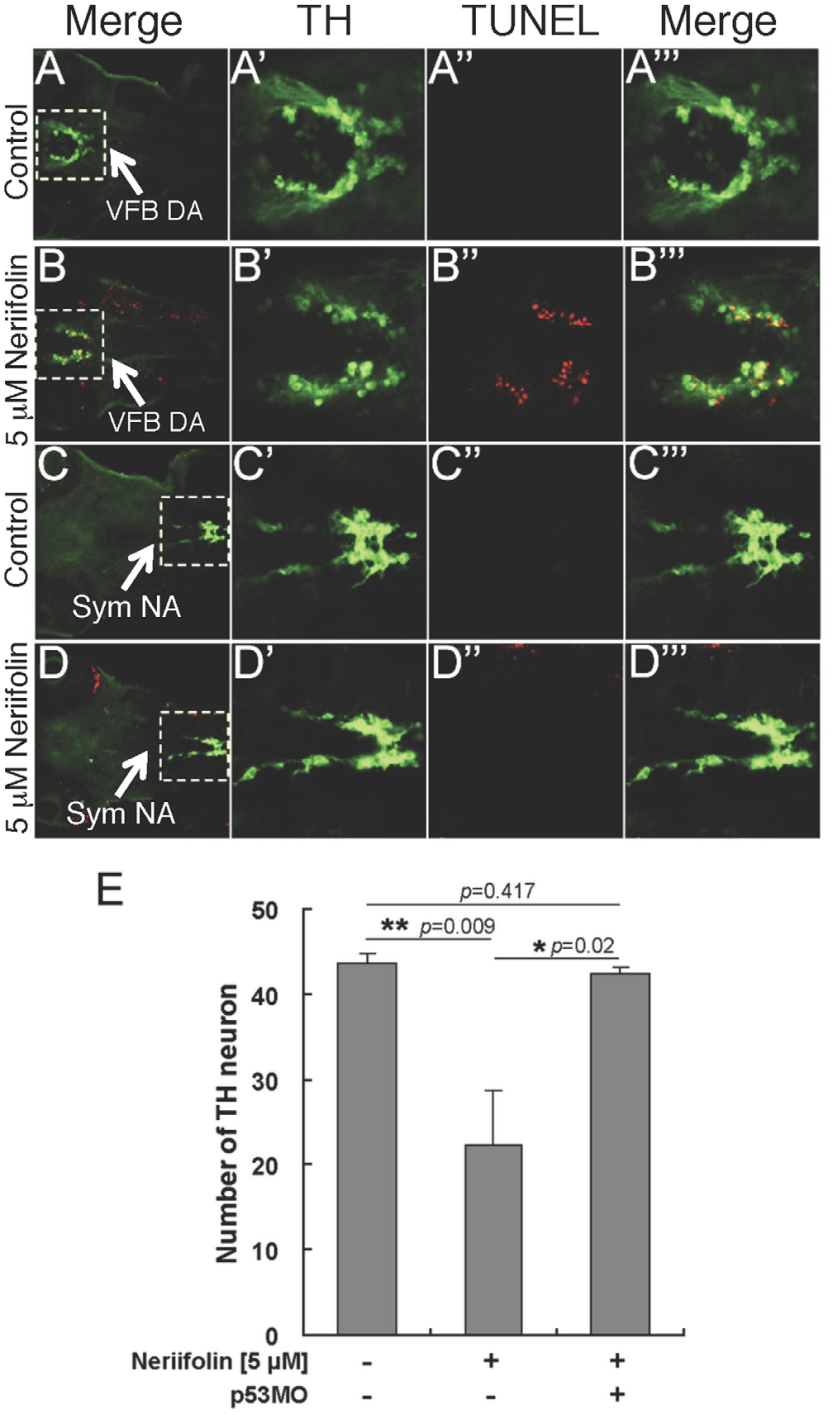
Neriifolin-induced DA neuronal death is apoptotic and requires p53. (A–D′″) Low (A–D) and high (A′–D′″) magnification views of VFB DA neurons in control (A-A′″) vs Neriifolin-treated embryos (B-B′″), and sympathetic (Sym) NA neurons in control (C-C′″) vs Neriifolin-treated embryos (D-D′″). Ventral views of 60 hpf embryos were shown. Neriifolin treatment was carried out from 24 hpf to 60 hpf. (E) Injection of the p53-MO into embryos at one-cell stage protected DA neurons from cell death induced by Neriifolin. Data are the averages ± SEM from 6 embryos in a single experiment that was repeated twice with similar results.

To gain molecular insight into Neriifolin-induced DA neuronal death, we investigated whether this process is dependent on p53, a tumor suppressor that integrates cellular stress signal and activates apoptosis [33]–[35]. We first determined whether the Neriifolin-induced DA neuronal death is apoptotic by performing TUNEL labeling. This analysis showed that DA neurons indeed are preferentially TUNEL^+^ upon treatment with Neriifolin (**Fig. 4A–B**). However, TUNEL staining was not solely restricted to DA neurons. Nevertheless, TH-positive sympathetic NA neurons, which were unaffected by Neriifolin, were TUNEL^−^, suggesting that not all neurons were equally affected by the drug (**Fig. 4C–D**). Next, we injected a well-established morpholino antisense oligonucleotide targeting p53 [36] (with over 150 publications in the pubMED employing this morpholino oligonucleotide) into one-cell stage wild-type embryos (thereof referred to as p53 morphants). Control or p53 morphants were treated with Neriifolin from 24 hpf to 72 hpf. Impairment of p53 activity exerted a complete protection of DA neurons (**Fig. 4E**): while significant difference existed between the DMSO control and Neriifolin group (*p* = 0.009), there was no difference between DMSO control group and Neriifolin/p53 morphant group (*p* = 0.417). This result indicates that Neriifolin-induced DA neuronal apoptosis requires p53.

### Impairment of DA neuronal survival by Neriifolin is evolutionarily conserved

**Figure 5:**
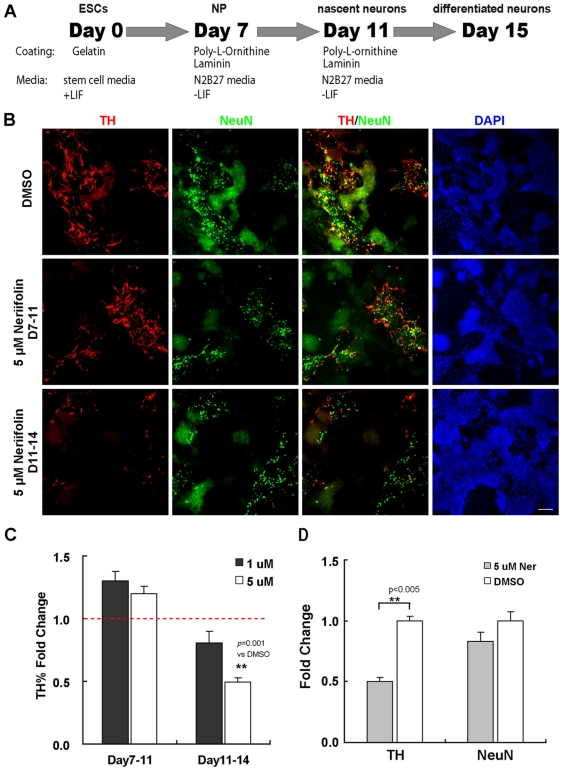
Neriifolin selectively impairs the survival of mammalian DA neurons derived from embryonic stem cells (ESCs). (A) Schematic diagram of the protocol used to induce DA neuron differentiation from mouse ESCs. (B) Representative views from wells with different treatment, showing the percentage of TH^+^ neurons (TH, a marker of DA neurons, in red) or all neurons (NeuN, a neuronal marker, in green) among total cells (DAPI). Compared to DMSO control (upper panel), those cells treated with Neriifolin from Day 7 to 11 (middle panels) showed no decrease in the percentage of TH^+^ neurons and all neurons; but those cells treated with Neriifolin from day 11 to 14 (lower panels) showed significant less percentage of DA neurons. (C) Quantification of the percentage of TH^+^ neurons among total cells. Fold change of treatment vs DMSO control was shown. (D) Quantification of fold change of TH^+^ neurons and all neurons. In both (C) and (D), data are the averages ± SEM of triplicates from a single experiment that was repeated 3 times with similar results.

To determine whether mammalian DA neurons are also vulnerable to Neriifolin, we tested the effect of the drug on neurons derived from mouse embryonic stem cells (mESCs). The mESC line E14 was induced to differentiate into neurons using the monolayer differentiation method [37] (**Fig. 5A**). After plating mESC cells in N2B27 media and withdrawal of LIF on Day 7, Nestin^+^ and Sox2^+^ neural progenitors began to emerge in the culture (**Fig. S1**). On Day 11, these progenitors differentiated into nascent neurons (positive for the pan-neuronal marker NeuN with a subset of them TH^+^). On Day 15, NeuN^+^ and TH^+^ neurons with more elaborated processes were detected in the culture, indicative of more differentiated status. Double labeling with midbrain DA neuronal markers and TH showed that many TH^+^ neurons were DA neurons of the midbrain characteristics (**Fig. S2**).

We treated cells with Neriifolin either from Day 7 to 11 (containing mostly neural progenitors and some nascent neurons) or from Day 11 to 14 (containing mostly differentiated neurons). The quantification was carried out using the InCell 2000 automated imaging and analysis software (**Fig. S3**). In agreement with the *in vivo* zebrafish data, only cells treated from Day 11 to 14 showed decreased TH^+^ neurons compared to vehicle controls (**Fig. 5B–C**), suggesting that Neriifolin impairs the survival rather than the differentiation of mammalian DA neurons. Moreover, the decrease of TH^+^ neurons was highly significant (*p*<0.005, compared to DMSO control) but the total number of neurons was not significantly affected compared to DMSO control (**Fig. 5D**), again suggesting an increased sensitivity of DA neurons to Neriifolin.

## Discussion

**Figure 6:**
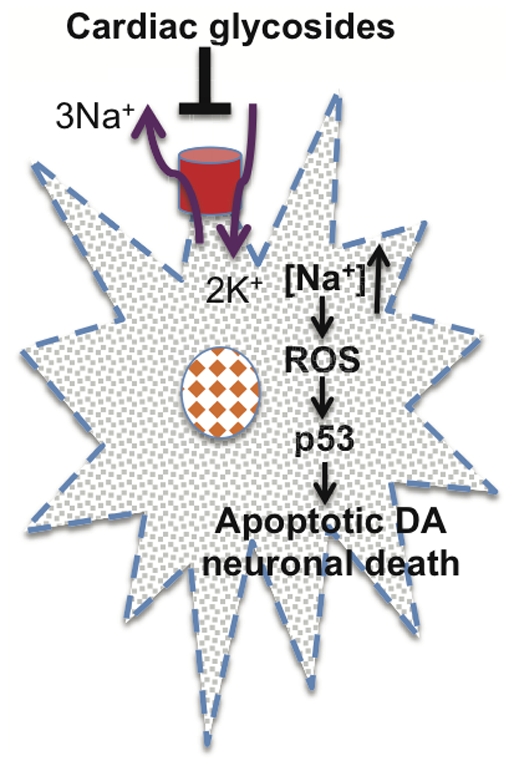
A schematic model showing the effect of cardiac glycosides on DA neuronal death. We propose that inhibition of Na+/K+ ATPase activity leads to increased intracellular sodium, which triggers the production of reactive oxygen species (ROS) and activation of p53-mediated apoptotic cell death pathway. Future biochemical experiments are needed to further test this model.

By screening for chemical modulators of DA neuron development or survival, we have uncovered a previously unknown vulnerability of DA neurons to cardiac glycosides and also identified strategies that protect DA neurons against the neurotoxicity of this class of medications. Based on our data, we propose the following model (**Fig. 6**) to explain Neriifolin-induced DA neuronal death: Inhibition of Na^+^/K^+^ ATPase activity by cardiac glycosides causes an ionic imbalance; in particular, increased intracellular Na^+^ levels aggravate oxidative stress. Through a yet unknown mechanism, this oxidative stress activates p53, which subsequently unleashes the apoptotic cell death pathway. Since DA neurons are known to be highly susceptible to oxidative stress due to intrinsic metabolic processes [38], this may explain the increased vulnerability of DA neurons to Neriifolin, as well as to other neurotoxic compounds such as MPTP [39]–[43] and rotenone [43], [44], which can selectively (in the case of MPTP) or non-selectively (in the case of rotenone) enter DA neurons and inhibit the activity of mitochondrial complex I of the electron transport chain [45]–[47].

While our data show that the sympathetic NA neurons are unaffected by Neriifolin in zebrafish and the overall neuronal numbers are not significantly reduced in Neriifolin-treated mESC cultures, it is unlikely that DA neurons are the only neuronal type affected by Neriifolin, given the broad Na^+^/K^+^ ATPase activity detected in the brain. Although such broad expression of Na^+^/K^+^ ATPase in neurons including DA neurons favors the notion that DA neuronal death is due to a cell autonomous mechanism, it also makes it difficult to rule out the non-cell autonomous contributions to Neriifolin-induced DA neuronal death.

In humans, most cardiac glycosides are known to cross both blood-brain barriers and placental barriers. Their adverse effects on the central nervous system have been reported that includes depression and psychosis but not PD [48], [49]. This may be due to the fact that DA neuron degeneration takes time to manifest and will likely result in symptoms at a much later time point after the offensive drug exposure. While it is not known whether drug concentrations experienced by embryonic and larval zebrafish are comparable to brain drug concentrations in human patients, it is worth pointing out that a single acute exposure of zebrafish to cardiac glycosides is sufficient to impair DA neuronal survival. Human patients usually take these drugs for longer periods of time, so even if they are on a lower dose, prolonged exposure to cardiac glycosides could affect DA neuronal homeostasis. Interestingly, the *atp1a3* gene in humans has been previously associated with the rapid-onset dystonia-parkinsonism (RDP), which is characterized by the rapid development of dystonic spasms, dysarthria, dysphagia, and parkinsonism [30]. However, it is presently not known whether brain DA neurons degenerate in this particular form of parkinsonism.

Our study has demonstrated the importance of zebrafish chemical screening for discovering previously unknown toxicity of therapeutic compounds as well as devising strategies to counteract their toxicity. Further chemical genetic studies of DA neuron development or survival, including characterizing other leads identified from this screen, and identifying more chemicals, not only employing wild-type, but also transgenic or pharmacological models with pre-existing DA neuron degeneration, promise to shed new light on the etiology of PD and development of neuroprotective therapeutics.

## Materials and Methods

### Ethics statement

All work followed the NIH guidelines and was approved by the University of California San Francisco Committee on Animal Research.

### Chemical screening and libraries

To screen for small molecules that can affect the differentiation and maintenance of DA neurons in zebrafish, wild-type embryos (in the AB genetic background) were collected and raised in Blue Egg Water (BEW, 0.12 g. CaSO4, 0.2 g. Instant Ocean, 30 microliters methylene blue in 1 L water) to 24 hpf. After removal of chorions, embryos were transferred into 96-well-deep-well polypropylene plates (Fisher, #12-566-120, n = 3 embryos per well) containing 10 µM chemicals (in 400 µl total volume). DMSO concentration was 1%. Embryos were treated with chemicals for 24–72 hours at 28°C. At the end of chemical treatment, embryos were observed under a dissecting microscope and abnormal morphologies were recorded, including the heart rate, cardiac edema, and overall body shape. Afterwards, embryos were transferred to a mesh-bottom-96-PCR plate for immunostaining with a polyclonal TH antibody (custom-made, 1∶1000), followed by visualization of DA and NA neurons under a fluorescent dissecting microscope (Leica).

Chemical libraries were provided by the UCSF Small Molecule Discovery Center (SMDC). Two libraries were screened: Microsource and Celera_IFLabs. The Microsource library contains 1520 compounds and the Celera library contains 3500 compounds.

### Immunostaining and confocal microscopy

TH immunostaining was carried out as previously described [50], [51]. TUNEL labeling was performed according to the manufacturer's protocol (Roche In situ Cell Death Detection Kit). Immunostained zebrafish embryos were mounted in glycerol and imaged using a Leica SP2 confocal microscope as a stack of roughly 100 optical sections taken approximately 1 µm apart in the z-dimension. This stack of images was imported into ImageJ software and average intensity projections were created for each zebrafish embryo.

### *In situ* hybridization

In situ hybridization was carried out as previously described [50].

### Plasmids, morpholinos, and microinjection

The *hsp70* promoter [52] was used to drive the human ATP1A3 WT OR cDNA (a generous gift from Dr. L. Ozelius). The plasmid DNA was micro-injected into one-cell stage wild-type embryos at a concentration of 40 ng/µl, together with 10 ng/µl transposase RNA to increase integration of DNA and decrease possible mosaic expression. At 22.5 hpf, embryos with normal morphology were heat-shocked at 37°C for 90 minutes. 24 hours after heat shock (around 48 hpf), de-chorioned embryos were transferred into freshly made embryo media (NaCl, 13.7 mM; KCl, 0.5 mM; Na_2_HPO_4_, 25 µM; KH_2_PO_4_, 44 µM; CaCl_2_, 1.3 mM; MgSO_4_, 1 mM; NaHCO_3_, 0.41 mM; pH 7.2), with 5 µM Neriifolin (DMSO final concentration 0.5%). Neriifolin treatment lasted from 48 hpf to 80 hpf. The embryos were collected and immunostained afterwards.

The sequence of this *p53* morpholino is: GCGCCATTGCTTTGCAAGAATTG, which targets the ATG of *p53* mRNA [36]. The standard control morpholino from Gene Tools INc. was used. Control or the *p53* morpholinos were injected into one-cell-stage embryos, which were raised in Blue Egg Water until 24 hours, then transferred into Embryo Medium in a 24-well-plate and treated with 5 µM Neriifolin. At 72 hpf, embryos were collected and fixed, then stained with anti-TH antibody.

### Mouse ESC culture and DA neuronal differentiation

Mouse embryonic stem cell line E14Tg2a was used [53]. E14 cells were cultured in GMEM media (Sigma, G5154) supplemented with glutamine, sodium pyruvate, 0.1 mM MEM non-essential amino acids, 10% (v/v) fetal bovine serum (characterized, Hyclone), beta-mercaptoethanol, and 500–1000 units per ml of leukocyte inhibitory factor (Chemicon ESG1107), on gelatinized cell culture surface without feeder cells.

To induce neuronal differentiation, the monolayer differentiation protocol developed by Ying et al was used [54]. Briefly, on Day 1, E14 cells were dissociated with Trypsin-EDTA (TE) into single cells and plated onto gelatinized cell culture dish at a density of 1.0×10^4^ cell/cm^2^ in N2B27 media. Cells were cultured in N2B27 media for 7 days (with media changes every other day) to differentiate ESCs to neural progenitors. On day 7, cells were dissociated with TE and re-plated onto poly-L-ornithine-laminin coated 96-well plates in N2B27 media. Media was changed every 2 days after re-plating. On Day 15, cells were fixed with PFA and immunostained with antibody for analysis the differentiation of TH neurons or all neurons. For chemical treatment, chemical was diluted into media on Day 8 or Day 11 and treatment lasted for 4 days, with media changes every other day.

Primary antibody for immunocytochemistry included: Rabbit-anti-TH, 1∶2000 (Millipore, AB152); Mouse-anti-NeuN, 1∶600 (Millipore, MAB377); Nurr1 (Santa Cruz, Sc-991), Lmx1a (a generous gift from Dr. M. S. German, UCSF). After immunostaining, images were taken using the automated imaging system InCell 2000 (GE). 20 views of images were taken for each well and for each view, images from 3 channels were taken (TH, NeuN plus DAPI). Image stacks were segmented and quantified using the software InCell developer. The percentage of TH neurons and all neurons were calculated accordingly.

## Supporting Information

Figure S1 **Characterization of neuronal progenitors in Day 7 mESC culture.** At this stage, most cells in culture were neuronal progenitors, as evidenced by the expression of Sox2 (top) and Nestin (bottom).
(TIF)

Figure S2 **Many TH^+^ cells in the mESC culture are of midbrain DA identity.** Top panels: midbrain DA markers Nurr1; middle panels: midbrain DA markers Lmx1a; bottome panels: the pan neuronal marker NeuN.
(TIF)

Figure S3 **Quantification using automated INCell imaging analysis software.** Representative images showing the segmentation method used in InCell Developer software (A). (B) Targets from different channels were segmented separately and the area of all targets from all the views in a well is summarized. TH% among total cells was calculated based on the areas from TH channel and DNA channels (Cal of TH). The fold change upon certain treatment (e.g. Neriifolin) was calculated by computing the ratio over the control (e.g. DMSO).
(TIF)
