# Supplementary figures and images for: Zebrafish Chemical Screening Reveals the Impairment of Dopaminergic Neuronal Survival by Cardiac Glycosides
Source: PLoS One. 2012 Apr 26;7(4):e35645. doi: 10.1371/journal.pone.0035645 (PMC3338518; doi:10.1371/journal.pone.0035645)

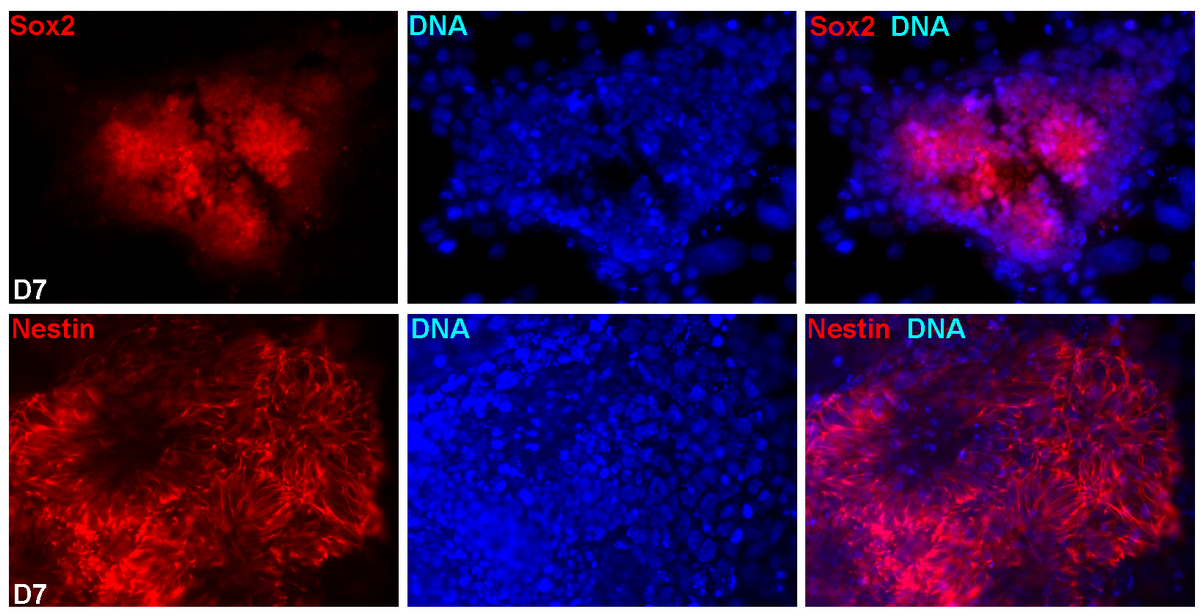

Supplement: Figure S1 — Characterization of neuronal progenitors in Day 7 mESC culture. At this stage, most cells in culture were neuronal progenitors, as evidenced by the expression of Sox2 (top) and Nestin (bottom). (TIF) [file pone.0035645.s001.tif]

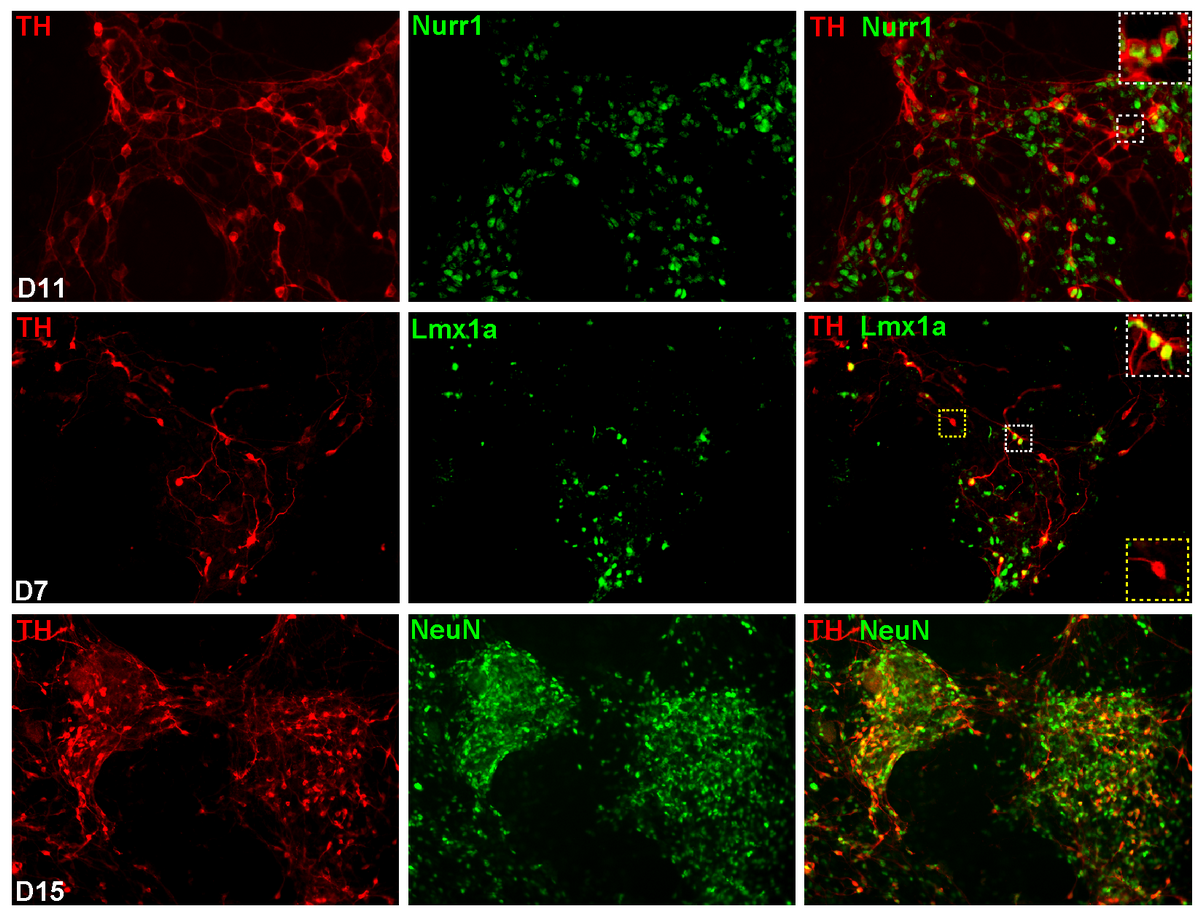

Supplement: Figure S2 — Many TH+ cells in the mESC culture are of midbrain DA identity. Top panels: midbrain DA markers Nurr1; middle panels: midbrain DA markers Lmx1a; bottome panels: the pan neuronal marker NeuN. (TIF) [file pone.0035645.s002.tif]

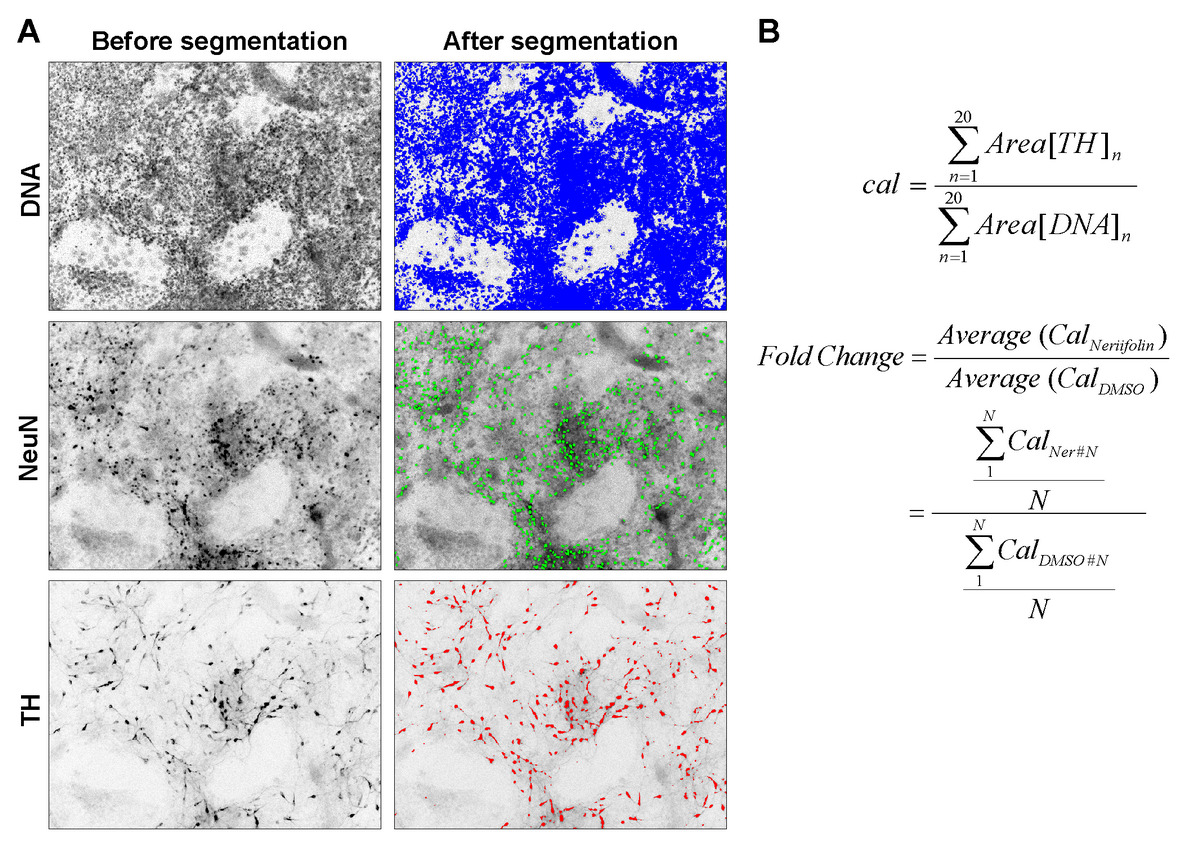

Supplement: Figure S3 — Quantification using automated INCell imaging analysis software. Representative images showing the segmentation method used in InCell Developer software (A). (B) Targets from different channels were segmented separately and the area of all targets from all the views in a well is summarized. TH% among total cells was calculated based on the areas from TH channel and DNA channels (Cal of TH). The fold change upon certain treatment (e.g. Neriifolin) was calculated by computing the ratio over the control (e.g. DMSO). (TIF) [file pone.0035645.s003.tif]
